# Assessing the Economic Viability of the Plastic Biorefinery Concept and Its Contribution to a More Circular Plastic Sector

**DOI:** 10.3390/polym13223883

**Published:** 2021-11-10

**Authors:** Megan Roux, Cristiano Varrone

**Affiliations:** Section for Sustainable Biotechnology, Department of Chemistry and BioScience, Aalborg University, A. C. Meyers Vænge 15, C2, 2450 Copenhagen, Denmark; meg.roux.jozi@gmail.com

**Keywords:** plastic biorefinery, plastic waste, upcycling, circular economy, PET, PEF, PTT

## Abstract

It is widely accepted that plastic waste is one of the most urgent environmental concerns the world is currently facing. The emergence of bio-based plastics provides an opportunity to reduce dependency on fossil fuels and transition to a more circular plastics economy. For polyethylene terephthalate (PET), one of the most prevalent plastics in packaging and textiles, two bio-based alternatives exist that are similar or superior in terms of material properties and recyclability. These are polyethylene furanoate (PEF) and polytrimethylene terephthalate (PTT). The overarching aim of this study was to examine the transition from fossil-based to renewable plastics, through the lens of PET upcycling into PEF and PTT. The process for the production of PEF and PTT from three waste feed streams was developed in the SuperPro Designer software and the economic viability assessed via a discounted cumulative cash flow (DCCF) analysis. A techno-economic analysis of the designed process revealed that the minimum selling price (MSP) of second generation-derived PEF and PTT is 3.13 USD/kg, and that utilities and the feedstock used for the production of 2,5-furandicarboxylic acid (FDCA) needed in PEF synthesis contributed the most to the process operating costs. The effect of recycling PEF and PTT through the process at three recycling rates (42%, 50% and 55%) was investigated and it was revealed that increased recycling could reduce the MSP of the 2G bio-plastics (by 48.5%) to 1.61 USD/kg. This demonstrates that the plastic biorefinery, together with increasing recycling rates, would have a beneficial effect on the economic viability of upcycled plastics.

## 1. Introduction

The projected amount of global plastic waste is 500 million tonnes by 2030, of which the majority is food packaging, comprising 60% of all coastal waste [1]. This has severe detrimental effects on the environment and human health. However, plastic is useful in a wide range of applications and results in lower CO_2_ emissions from transportation when compared to glass [2]. A system for the production and use of plastics that is more circular in nature-namely the circular economy-will result in a decrease in plastic production and waste [3]. In this system, the majority of plastic should be reusable or recyclable. However, the recycling of plastic is hindered by several barriers, including high collection costs, a wide range of polymers with different properties, and the fact that plastic products are often designed with functionality in mind instead of end-of-life scenarios [4]. Recycling plastic is also not considered to be economically viable as a result of virgin plastic being cheaper than recycled plastic, and a lack of market demand for recycled plastic [4]. In fact, it is estimated that 95% of the value of plastic packaging material is lost already after the first use [5].

Mechanical recycling, which is the most common recycling method employed currently, results in a loss of plastic molecular mass, and thus, the number of times an item of plastic can be recycled mechanically is limited [6]. Additionally, large fractions of mixed plastic waste and multilayer materials cannot be mechanically recycled [7]. In addition to limiting the amount of plastic produced, the resources for the production of new plastics should be renewable so as to reduce dependency on non-renewable resources such as fossil fuels [5], as it is expected that plastic’s contribution to global oil consumption will increase from the current 4–8% to 20% in 2050 [2]. The production of plastics made from renewable resources, bio-based plastics, is expected to increase from 2.11 million tonnes in 2020 to 2.87 million tonnes by 2025 [8]. In addition, the EU is increasing investment in bioplastics and aims to replace 30% of fossil-based plastic food packaging with bioplastic [9]. PET is a fossil-based plastic that is widely used in packaging and textiles, and it is also one of the most recycled plastics [10], with an estimated production of 70 million tonnes per annum [11]. Due to the depletion of non-renewable resources, such as the petroleum used to make conventional plastics, there is increasing focus on plastics made from renewable material [12]. It would, therefore, be interesting to identify bio-based polymers, with similar properties to PET, which have the potential to substitute it. Polyethylene furanoate (PEF) is a bio-based plastic that is considered to be the closest to PET with respect to thermal and mechanical properties. PEF is considered a superior polymer to PET for packaging, as a result of a higher glass transition temperature, T_g_, when compared to PET (82–87 °C for PEF and 71–75 °C for PET) [13]. In addition, PEF has several properties that make it superior to PET for the production of plastic bottles. The lower melting temperature, T_m_, of PEF (210–215 °C) compared to PET (246 °C) makes extrusion and blow moulding of the material easier. The Young’s modulus and strength of PEF is higher than for PET, which allows for thinner bottle walls [13] and a more resilient material [14]. Finally, PEF has excellent O_2_ and CO_2_ gas barrier properties, with PEF being 31 times less permeable to carbon dioxide than PET is. As with PET, PEF has a high thermal stability up to around 350 °C [14]. Eerhart et al. [15] compared the energy use and greenhouse gas (GHG) emissions for PEF and PET production, and found that completely replacing PET plastic bottles with bio-based PEF ones can save between 440 and 520 PJ of non-renewable energy use, and can result in a reduction in GHG emissions of 20–35 Mt of CO_2_ equivalent. Thus, PEF seems to represent an excellent example of a bio-based plastic that can replace PET in the food packaging sector as part of the transition to a circular plastics economy.

As mentioned above, PET is also widely used in the textile industry. PTT can be a bio-based alternative to PET that can be used in textiles and potentially also for packaging. PTT has a T_g_ of 50 °C and T_m_ of 228 °C [16] and displays excellent properties that make it superior to PET in fibre applications. The polymer can be dyed at higher temperatures (100 °C) than PET, which simplifies the dyeing process. PTT fabric that has been dyed has a better colour fastness and shows deeper shades than PET fabric [16].

The plastic is made similarly to PET, by polymerisation with terephthalic acid (PTA) and 1,3-propane diol (PDO) in place of EG. This means that it could easily be produced in already existing PET production sites [17]; moreover, the overall PTT polymerisation process is more energy efficient than for PET, leading to lower CO_2_ emissions [18]. Advances in cost-effective bio-based PDO production as well as the refinement of continuous polymerisation processes have allowed for the economical production of high-quality PTT, resulting in the commercialisation of the polymer by DuPont and by Shell. PTT is also known by its commercial name, Sorona^®^ (DuPont) [16], or Corterra (Shell). Overall, the energy efficiency of the PTT production process is greater than for PET production, as PTT polymerisation and downstream processing requires less energy than for PET [16]. Moreover, PDO can be produced through bioconversion of waste streams, such as biodiesel-derived crude glycerol [19]. Notably, PEF was shown to be easier to biodegrade and depolymerize than PET [15]. This represents a key element for a more circular plastic industry, since an efficient and easier depolymerization potentially allows for infinite recycling. Until now, very few studies have investigated PTT biodegradation, but it was reported that PTT can be enzymatically hydrolysed using diverse enzymes also used for PET depolymerisation [20]. Moreover, the longer (and uneven) diol chain and lower glass transition of PTT should, in principle, facilitate degradation compared to PET [21]. Could we then imagine using post-consumer PET waste as a source for more carbon-neutral substitutes, such as PEF and PTT?

**Figure 1:**
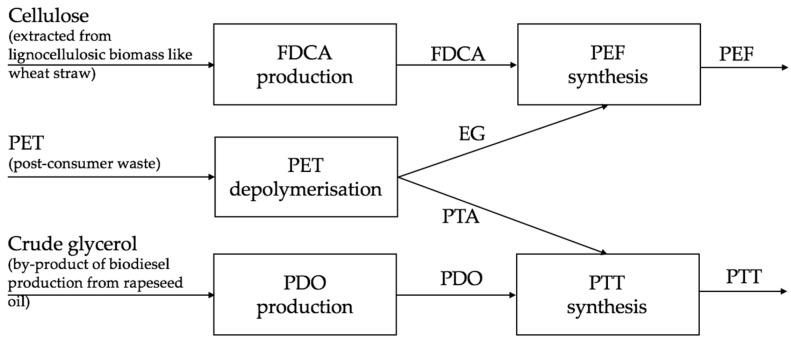
Block flow diagram showing the concept and material flow of the PET plastic biorefinery: three waste streams (biodiesel-derived crude glycerol, post-consumer PET and lignocellulosic biomass-derived cellulose) can be used to synthesise PEF and PTT.

When depolymerised, PET forms EG and PTA. These monomers can be used to make a range of materials, including new plastics. The synthesis of PEF requires EG, and the synthesis of PTT requires PTA. Thus, PET waste can be turned into valuable, renewable and bio-based plastics with improved properties, which is, in essence, the concept of biorefinery and bio-upcycling. In this sense, we might define this process as a ‘plastic biorefinery’ (Figure 1). The proposed basic steps involved in the transformation of PET waste into PEF and PTT are: PET depolymerisation, PEF synthesis (with the addition of bio-based FDCA), and PTT synthesis (with the addition of bio-based PDO).

The present work represents a conceptual case study to assess the contribution and economic viability of a ‘plastic biorefinery’ to the transition towards a more sustainable and circular plastic industry, through the valorisation of existing plastic waste streams. This novel approach proposes the integration of biochemical recycling of fossil-based plastic waste together with (2G) bio-based building blocks to produce more carbon-neutral polymers that are easier to recycle.

## 2. Materials and Methods

### 2.1. Simulation and Assumptions

The plastic biorefinery concept was designed from established processes found in the literature. From these established processes, information regarding unit processes (reactor type, etc.) and conditions (temperature, pressure, residence time, etc.), and experimental parameters (conversions, efficiencies, yields, etc.) were extracted. The mass flows were calculated using mass balances and then implemented in the SuperPro Designer software (version 9.5). Table 1, Table 2, Table 3 and Table 4 in Section 2.2 detail the most important parameters and flow rates. Continuous operation was chosen with a time on stream of 7920 h per annum. The processing rate is 68,000 tonnes per annum (tpa) of post-consumer waste PET. It is assumed that this PET is the residual plastic that is not recycled [11].

### 2.2. Process Design

**Figure 2:**
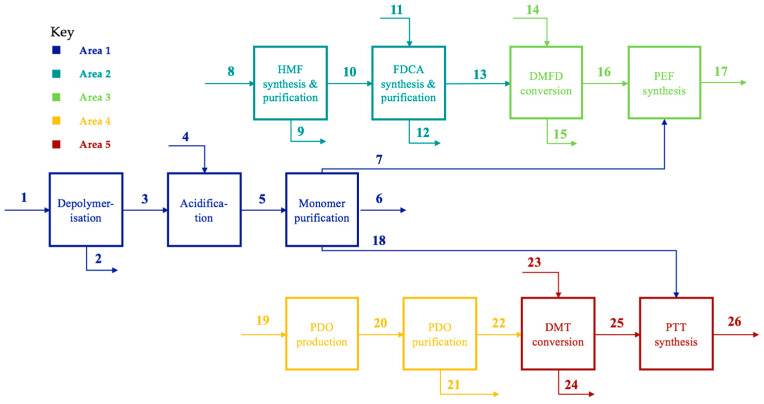
Expanded block flow diagram of the plastic biorefinery concept. Area 1: PET depolymerisation and purification of monomers, Area 2: FDCA production from cellulose, (3) PEF synthesis, (4) PDO production from crude glycerol, and (5) PTT synthesis.

The block flow diagram in Figure 1 describes the overall concept of the ‘plastic biorefinery’ in which post-consumer waste PET is upcycled into PEF and PTT. A detailed diagram of the process is shown here in Figure 2, representing the 12 main operations and the 28 streams associated with it. The process is divided into five principal areas. In Area 1, PET is enzymatically depolymerised into EG (stream 7) and PTA (stream 18), and these monomers are purified. Sodium sulfate (Na_2_SO_4_) is also formed and is sold (stream 6). In Area 2, FDCA is produced from lignocellulosic biomass-derived cellulose (stream 8), via the formation of 5-hydroxymethylfurfural (HMF). In Area 3, FDCA from Area 2 is converted to its dimethyl ester form (DMFD) before being added to EG from Area 1 to synthesise PEF (stream 17). In Area 4, PDO is formed from biodiesel-derived crude glycerol (stream 19) and purified. In Area 5, PTA is converted to its dimethyl ester form (DMT) and then used with PDO from Area 4 to synthesise PTT (stream 26).

The specifics of this process are described further in this Section 2.2.1, Section 2.2.2, Section 2.2.3, Section 2.2.4 and Section 2.2.5, and the flow rates for the streams shown in the figure can be found in Table S5a,b in the electronic Supplementary Materials. The full process, as modelled in the software, is shown in Figure S1a–e in the electronic Supplementary Materials. 

#### 2.2.1. Area 1: PET Depolymerisation

In Area 1, PET is enzymatically depolymerised, and the resulting monomers are purified for use in Areas 3 and 5. The PET depolymerisation process was designed based on the latest and most advanced process found in the literature [11], developed by Carbios and the University of Toulouse. The purification of the monomers was taken from the process described by Ügdüler et al. [10]. PET is thus depolymerised using a leaf-compost cutinase (LCC) enzyme in a batch reactor, forming EG and sodium terephthalate (Na_2_TP). The residual PET is removed by filtration and pigment is removed by adsorption. The Na_2_ TP is then acidified with sulfuric acid (Na_2_SO_4_) to form PTA and sodium sulfate (Na_2_SO_4_). Finally, PTA is purified from this stream by filtration and crystallisation. EG is then removed by flash separation and distillation, and the formed Na_2_SO_4_ can be sold. 

#### 2.2.2. Area 2: FDCA Synthesis

FDCA synthesis was modelled according to the process described by Kim et al. [22]. In this process, cellulose is directly dehydrated to 5-hydroxymethylfurfural (HMF) using a tetrahydrofuran (THF) solvent and Na_2_SO_4_ catalyst. Lime is added to neutralise the acid, after which THF is recovered by distillation and recycled in the process. Humins and other by-products are removed by activated carbon adsorption, and HMF is converted to FDCA using gamma-valerolactone (GVL) as a solvent and Pt/C as a catalyst. FDCA is purified by solid–liquid separation and GVL is recycled in the process. Levulinic acid (LA), formed as a by-product in HMF production, is used to synthesise GVL to replace that which was lost in the FDCA reactor.

#### 2.2.3. Area 3: PEF Synthesis

PEF can be synthesised by a number of means, although the process in this study is based on transesterification, as described by Kasmi et al. [18]. First, FDCA is esterified to the dimethyl ester of FDCA (DMFD). This is conducted by reacting FDCA with methanol, where excess methanol is removed by distillation and recycled in the process. DMFD is then cooled and purified by microfiltration and crystallisation. DMFD and EG from Area 1 are then mixed together in a pasting unit before polymerisation in three stages on a tetrabutyl titanate (TBT) catalyst: transesterification, pre-polymerisation and polycondensation, during which PEF is formed at high temperatures and vacuum conditions. Methanol is formed as a by-product and is removed from excess EG by distillation, and both components are recycled in the process. The PEF molecular weight is further increased by extruding, cooling and cutting the polymer before a final polymerisation step, solid state polymerisation (SSP), at high temperature and vacuum conditions.

#### 2.2.4. Area 4: PDO Synthesis

In Area 4, the PDO required for PTT synthesis with PTA is formed via the fermentation of crude glycerol obtained as a by-product from biodiesel production, following the highly efficient bioconversion process described by Chatzifragkou et al. [23]. In this section of the process (Area 4), *Clostridium butyricum* cells are cultivated in shake flasks before being used in a series of fermentation reactors of increasing size, to which crude glycerol and fermentation medium is added. The fermentation under non-sterile conditions occurs in a fed-batch process, and results in the production of 67.9 g/L [23]. The PDO formed in these fermentation reactors is purified by filtration, ion exchange and evaporation, as described by Petrides et al. [24].

#### 2.2.5. Area 5: PTT Synthesis

Considering the similarities in properties and process routes between PTT and PET, PTT synthesis modelling was based on the PET synthesis process. In this Area, PTA is esterified to dimethyl phthalate (DMT) in place of FDCA to DMFD conversion.

### 2.3. Cost Estimations and Cash-Flow Analysis

Capital and operating cost estimations were extracted from the model, and a discounted cumulative cash flow (DCCF) analysis was performed using chemical engineering heuristics [25] and following the procedure set up by a National Renewable Energy Laboratory (NREL) technical report [26], with factors localised to the Danish context. Detailed information regarding the major inputs to the model and DCCF analysis can be found in the electronic Supplementary Materials (Tables S1 and S2, respectively). Further descriptions regarding the basic calculations used by SuperPro Designer to perform the economic evaluation are available in [27].

## 3. Results and Discussion

The designed process resulted in the production of 59,000 and 53,000 tpa PEF and PTT, respectively, from waste streams of 68,000 tpa PET, 132,000 tpa cellulose and 65,000 tpa crude glycerol.

The MSP of PEF and PTT was calculated using a DCCF analysis, and this was compared to the selling price (SP) of conventional PET. The capital and operating costs of the process (Table S3) were then examined to highlight any bottlenecks or areas for potential optimisation, and a sensitivity analysis was performed to determine the parameters that could have significant effects on the process.

### 3.1. Minimum Selling Price

More specifically, a DCCF analysis was used to estimate the MSP of PEF and PTT required for a net present value (NPV) of zero, after ten years of operation. The NPV is the DCCF value after a defined period of time, and when the NPV is zero it indicates the break-even point. The MSP is calculated as the minimum price at which a product must be sold so as to make the process profitable (where the NPV is zero) after a defined period of time.

Two scenarios were compared: (1) producing FDCA from a 2G cellulose feedstock, and (2) directly purchasing FDCA, at a price of 1.90 USD/kg [12]. For the latter, it is assumed that the FDCA is produced from fructose or high fructose corn syrup (HFCS), as these are the most common feedstocks for FDCA production at present [12,13]. HFCS was not used as the feedstock for Scenario 1 in this study as it is a first-generation (1G) feedstock, which could result in competition with food production. Second generation (2G) feedstocks—such as waste streams from other sectors—are preferred. Details about the DCCF of Scenario 1 are provided in Table S4, while the most important process streams (in kgh) are shown in Table S5.

In order to assess the minimum selling price, the PEF and PTT selling price was set as 2.70 USD/kg, which is the estimated production cost of PEF [28].

The MSPs of Scenario 1 (where FDCA is produced on-site from cellulose) and Scenario 2 (where FDCA is purchased directly) are 3.13 and 2.34 USD/kg, respectively. When compared to the selling price of PET, which is estimated between 0.725 and 0.950 USD/kg [29], it is seen that these plastics are not directly economically competitive with PET when produced via the designed process. On the other hand, it is expected that the PEF produced on an industrial scale (100 ktpa) by Avantium will have a market price of between 4.00 and 5.00 EUR/kg (4.62–5.77 USD/kg) [30], and Sorona^®^ (PTT by DuPont) is sold for 4.00 EUR/kg [31]. Thus, the PEF and PTT produced via this process could be economically competitive on the market, demonstrating the potential of the plastic biorefinery. In addition, these market estimates for commercial PEF and PTT indicate that there is a market for these more expensive bio-based alternatives to PET, even though it is questionable whether they would be able to substitute PET on a larger scale at the moment.

Table 5 shows that Scenario 2, where HFCS-derived FDCA is purchased and used for PEF synthesis, is more profitable. However, as mentioned above, this would make use of first-generation feedstocks. In general, and as part of the circular economy approach, the utilisation of residual streams is preferred so as to not compete with food production, while avoiding accumulation (and mismanagement) of waste.

The payback period (PBP) for both scenarios was also determined. The PBP is calculated as the fixed capital investment over the average cash flow. This is an indication of how long it would take for investors to earn back their investment. Obviously, the lowest possible PBP is desired, and investors may be unwilling to invest if the projected PBP is decades long, which further highlights that Scenario 2 is the preferred solution from an economic perspective.

However, as mentioned, the valorisation of waste streams is preferred in a biorefinery and circular economy concept. Therefore, in the next section, the capital and operating costs of Scenario 1 will be further investigated to identify areas of the process that can be improved to make PEF and PTT production from waste streams more economically viable.

### 3.2. Capital and Operating Costs in Scenario 1

**Figure 3:**
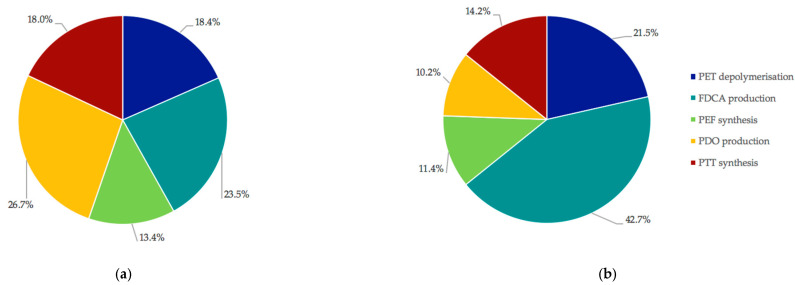
Distribution of capital costs (**a**) and operating costs (**b**) between the process Areas of Scenario 1. PET depolymerization = Area 1; FDCA production = Area 2; PEF synthesis = Area 3; PDO production = Area 4; PTT synthesis = Area 5.

To understand the process hot-spots and identify areas of optimisation, so as to improve the profitability of the 2G-based process and make PEF and PTT more economically competitive with PET, Scenario 1 was further examined (Figure 3).

**Figure 4:**
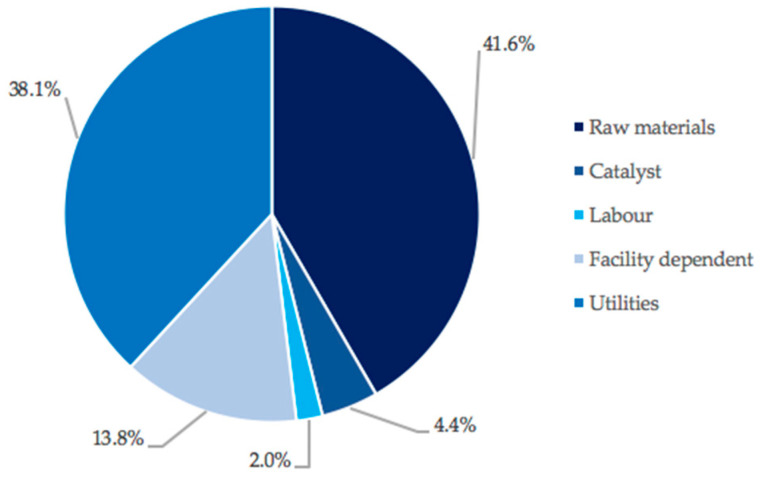
Breakdown of Area 2 operating expenses.

While the capital costs are rather evenly distributed among all areas, it is clearly seen that Area 2 (FDCA production) contributes disproportionately to the operating costs. Consequently, the operating costs within Area 2 were further examined, and are shown in Figure 4.

As it is seen, raw materials and utilities contributed most to the Area operating costs (with 41.6%). Within raw materials, cellulose contributed 94%. This high cost of the feedstock is likely due to the processing of lignocellulosic material required to extract the cellulose. This can represent a challenge when using second generation feedstocks, as the lower cost of the initial feedstock does not always overcome the cost associated with processing the complex/recalcitrant material into a usable form.

On the other hand, FDCA can be produced from a number of feedstocks and via a number of process routes [13]. For example, FDCA can be produced from pectins—which can be extracted from residual streams such as sugar beet pulp and citrus peels—via the formation of 2-formyl-5-furoic esters instead of via HMF [32]. Another study demonstrated that HMF can be produced from the simultaneous conversion of glucose and xylose to HMF and furfural [33], thus increasing the overall HMF yield and allowing for more of the feedstock to be exploited. For example, using a wheat straw feedstock, which contains both glucose and xylose, with this method, means that almost all of the substrate could be used instead of only the glucose-containing part of the feedstock. However, these routes have yet to be assessed on an industrial scale and were, therefore, not used in this study, but could provide an alternative to the challenge of sustainable and viable FDCA production in the future.

Last but not least, Figure 4 also shows that utilities constituted the second largest contribution to Area 2 operating costs (with 38.1%). This was mostly due to the requirement of high-pressure steam for the cellulose conversion reactor, where high temperatures (483 K) are required. This contribution could be mitigated by heat integration across the process (because the re-use of heat generated inside the process can reduce the need for external energy consumption, such as electricity and steam [34]) or the development of efficient biological processes that operate at milder conditions.

### 3.3. Sensitivity Analysis

**Figure 5:**
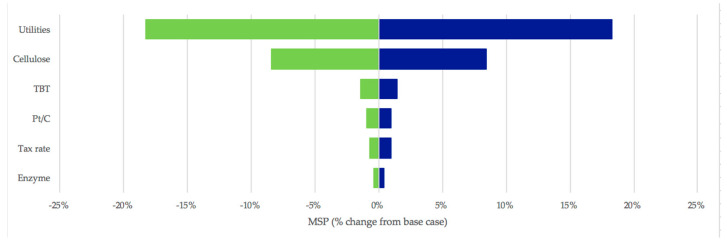
Sensitivity analysis of Scenario 1. TBT = tetrabutyl titanate, the catalyst used in PEF and PTT synthesis; Pt/C = the catalyst used in cellulose−to−HMF conversion.

A sensitivity analysis of a process reveals which factors affect the process the most. Figure 5 shows a sensitivity analysis for the process, used to assess the impact that possible changes (within a certain range) of such key factors would have on the profitability of the process. For this purpose, cost-driving parameters such as the price of cellulose, catalysts, enzymes, and utilities, as well as the tax rate, were changed to 50% below and above the base-case values.

As can be seen in Figure 5, the process is most sensitive to changes in the price of utilities, which contributes to 35% of the overall operating costs of the process. Increasing the overall utility costs by 50% would result in an MSP of 3.70 USD/kg (instead of 3.13 USD/kg). To minimise the contribution of utilities to the process, plant-wide heat integration should be implemented and could result in an MSP of 2.58 USD/kg (when utilities consumption is reduced by half). This would have both economic and environmental benefits.

As expected from the breakdown of operating costs over Area 2, the price of cellulose also has a significant impact on the process. The catalysts used in PEF and PTT synthesis (Areas 3 and 5) and in the cellulose-to-HMF conversion (Area 2) have the next greatest impacts on the process.

### 3.4. The Contribution of Policies to a More Circular Plastic Sector 

**Figure 6:**
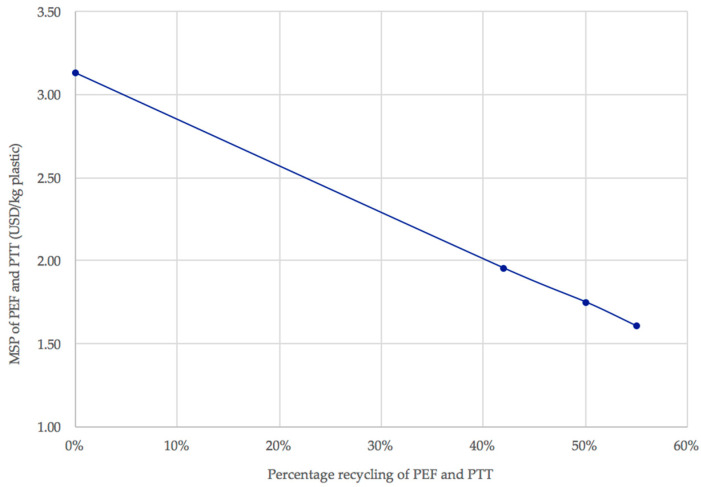
The MSP, in USD/kg, of PEF and PTT with changing recycling rates (42%, 50% and 55%) of PEF and PTT.

A recent study by Fernando Foncillas et al. [35] underlined the importance of public subsidies in the development of economically viable biorefineries. Clearly, dedicated subsidies could facilitate the transition towards 2G plastic biorefineries as well, thus supporting the bio-based economy. Moreover, policy targets and increasing recycling rates may also affect the economic viability of PEF and PTT. It is, therefore, important to investigate the potential impact of such policies. In 2015, the European Commission proposed that at least 55% of all plastic packaging should be recycled by 2025 [36], although it was estimated that only 42% of plastic was recycled in the European Union in 2017 [37]. How would those recycling rates affect the plastic biorefinery economy? Figure 6 shows different recycling scenarios for the upcycled PEF and PTT (0%, 42%, 50% and 55%) and their effect on the MSP.

It was assumed in these scenarios that PEF and PTT are recycled and, therefore, less FDCA and PDO are required. Thus, the following assumptions were made:

- PEF and PTT are depolymerised and the monomers purified in the same way as PET;
- Less PET is produced (and therefore recycled) as PEF and PTT replace it;
- Area 1 functions as normal;
- Area 2 and 4 have less throughput (adjusted to meet the EG and PTA output from PET depolymerisation);
- Area 3 and 5 have slightly less throughput.

Implementing these into the model, it can be seen that the increasing of recycling has a significant effect with a clear trend (Figure 6).

As seen from Figure 6, recycling PEF and PTT through the process reduces the MSP of these plastics, indicating that the implementation of such policies would have a beneficial effect on the economic viability of the upcycled 2G bio-based plastics. In fact, at 55% recycling, the MSP of the plastics reaches 1.61 USD/kg (48.5% lower than without recycling). While this is still higher than the selling price of PET, this nonetheless provides a useful strategy for making renewable plastics more competitive on the market. Notably, recycled PET has still a higher cost than the virgin one.

In addition to economic viability, it is also important to consider the environmental impacts of recycling. In fact, plastic production and incineration lead to a global production of 600 million tonnes of CO_2_ per year [1]. The present study does not include a life cycle analysis (LCA), which is imperative to understanding the actual benefits of this concept. However, it was estimated that the potential annual energy savings obtained from recycling all of the plastic waste produced globally would be equivalent to 3.5 billion barrels of oil per year. This would translate into 1 million cars off the road for each million tonnes of plastic recycled [2], thus clearly showing the CO_2_ benefit of increased plastic recycling, which can also help reduce our dependence on fossil fuels.

Last but not least, the impacts of this process should be compared to other means of re-utilising the PET, as well as a scenario in which the PET is recycled into itself. Further investigation into the utilisation or valorisation of the process waste streams should also be investigated. Moreover, assumptions should be carefully evaluated against full-scale real-case scenarios, and data added into the model to obtain more accurate estimations.

## 4. Conclusions

This study investigated the initial profitability of a conceptual process wherein post-consumer waste PET was depolymerised and upcycled into bio-based and renewable plastics, namely PEF and PTT, which could technically be used to replace PET in the food packaging and textile industries. A techno-economic analysis of this process revealed that the minimum selling price (MSP) of PEF and PTT produced via this process is 3.13 USD/kg when using second generation feedstocks, and 2.34 USD/kg when purchasing FDCA directly. This is around 2–3 times the current PET selling price, indicating that a complete substitution of PET is far from being economically feasible (with the current technology). On the other hand, when compared to the estimated selling prices of commercial PEF and PTT (up to 4.60 USD/kg), the plastics produced via the designed process in this study are economically competitive, thus demonstrating the potential of the plastic biorefinery concept.

Notably, adopting the European targets for recycling would have an important beneficial effect on the economic viability of the plastic biorefinery, further decreasing the MSP of the 2G bioplastics to 1.61 USD/kg and making these new plastics more competitive on the market. This should provide effective motivation for increasing recycling rates and suggests that the plastic biorefinery concept could play an important role in the transition to a more bio-based and circular plastics sector.

## Figures and Tables

**Table 1:** Specifications for mass balances and conversions in Area 1.

Parameter	Value	Source
PET flow rate	67,853 tpa	Calculated
Enzyme loading	0.2 wt.%	[11]
PET conversion	90.0%	[11]
PTA yield	86.3%	[11]

**Table 2:** Specifications for mass balances and conversions in Area 2.

Parameter	Value	Source
Cellulose flow rate	131,900 tpa	Calculated
Cellulose loading	3.0 wt. %	[22]
HMF yield	42 mol %	[22]
FDCA yield	93.6 mol %	[22]
FDCA recovery	95%	[22]

**Table 3:** Specifications for mass balances and conversions in Area 3.

Parameter	Value	Source
FDCA conversion	83%	[18]
PEF purity	99.4%	Calculated
PEF flow rate	59,150 tpa	Calculated

**Table 4:** Specifications for mass balances and conversions in Area 4.

Parameter	Value	Source
Crude glycerol flow rate	64,550 tpa	Calculated
Crude glycerol purity	81%	[23]
Crude glycerol in feed	75%	[23]
Crude glycerol flow rate	64,550 tpa	[23]
PDO purity	99.3%	Calculated

**Table 5:** Selling price (SP), payback period (PBP) and MSP of Scenario 1 and 2.

	Scenario 1	Scenario 2
SP (USD/kg)	2.70	2.70
PBP (years)	23.3	5.15
MSP (USD/kg)	3.13	2.34

## Data Availability

The data presented in this study are available on request from the corresponding author.

## Supplementary Materials

The following are available online at https://www.mdpi.com/article/10.3390/polym13223883/s1, Table S1: Major cost inputs; Table S2: Inputs to the DCCF analysis; Table S3: Summary of capital and operating costs; Table S4: DCCF of Scenario 1; Table S5a,b: Stream table showing most important process streams relating to Figure 2 in the main report. Flows are in kg/h; Figure S1a: Process flow diagram of Area 1; Figure S1b: Process flow diagram of Area 2; Figure S1c: Process flow diagram of Area 3; Figure S1d: Process flow diagram of Area 4; Figure S1e: Process flow diagram of Area 5.

## Abbreviations

DCCF	Discounted cumulative cash flow
FDCA	2,5-furandicarboxylic acid
HMF	5-hydroxymethyl furfural
MSP	Minimum selling price
PDO	1,3-propane diol
PEF	Polyethylene furanoate
PET	Polyethylene terephthalate
PTT	Polytrimethylene terephthalate
