# Supplementary material for: Assessing the Economic Viability of the Plastic Biorefinery Concept and Its Contribution to a More Circular Plastic Sector
Source: Polymers (Basel). 2021 Nov 10;13(22):3883. doi: 10.3390/polym13223883 (PMC8617788; doi:10.3390/polym13223883)
Supplement: Supplementary file 1 [file polymers-13-03883-s001.zip › polymers-1454107-supplementary.pdf]

Supplementary information

Assessing the economic viability of the plastic biorefinery and its contribution to a more circular plastic sector

Megan Roux and Cristiano Varrone

Table S1: Major cost inputs to the model

Component	Cost (USD/kg)	Reference
**Raw materials**		
LCC enzyme	25.0	[1]
NaOH	0.32	[2]
H_2SO_4	0.07	[2]
THF	1.53	[3]
O2	0.04	[3]
Cellulose	0.45	[3]
Crude glycerol	0.14	[4]
C. butirycum culture	213	[5]
PDO pre-culture medium	0.007	[2]
PDO fermentation broth	0.004	[2]
Methanol	0.24	[2]
Water	1.33	[6]
**Catalysts***		
Pt/C	194	[3]
RuSn4/C	539	[3]
TBT	150	[7]
**Utilities**		
Cooling water	0.00005	[2]
Chilled water	0.0004	[2]
Steam	0.012	[2]

High pressure steam	0.02	[2]
Electricity**	0.1	[2]
**Revenues**		
Na_2SO_4	1.00	[2]
Activated carbon	1.20	[3]
PEF	2.70	Assumed
PTT	2.70	Assumed

*Catalyst life span is assumed 6 months, with 10 % replacement at 20 % of the cost [3].

**In USD/kW-h

Table S2: Inputs to the DCCF analysis

Parameter	Value	Reference
Cost of PET pre-treatment (USD/kg PET)	277	[8]
Cost of labour (USD/h)	34.5	[9]
Construction period (years)	3	[10]
1st year expenditures (%)	8.0	[10]
2nd year expenditures (%)	60	[10]
3rd year expenditures (%)	32	[10]
Tax rate (%)	22	[11]
Depreciation (years)*	5.0	[12]
Working capital (% of fixed capital)	15	[12]
Start-up time (years)	0.5	[10]
Revenue during start-up (% of normal)	50	[10]
Operating costs during start-up (% of normal)	75	[10]
Discount rate (%)	10	[10]

*Straight-line depreciation over 5 years

Table S3: Summary of capital and operating costs, and revenues.

Fixed capital (USD)	498 379 707
Working capital (USD)	74 810 956
Total capital investment (USD)	573 550 663
Revenues (USD/year)	391 411 846
Operating costs (USD/year)	367 323 069

Table S4: DCCF of Scenario 1 in USD

	2022	2023	2024	2025	2026	2027	2028	2029	2030	2031	2032	2033	2034	2035
Revenue				195,705,923	391,411,846	391,411,846	391,411,846	391,411,846	391,411,846	391,411,846	391,411,846	391,411,846	391,411,846	391,411,846
Operating Costs				275,492,302	367,323,069	367,323,069	367,323,069	367,323,069	367,323,069	367,323,069	367,323,069	367,323,069	367,323,069	367,323,069
Gross Profit				-79,786,379	24,088,777	24,088,777	24,088,777	24,088,777	24,088,777	24,088,777	24,088,777	24,088,777	24,088,777	24,088,777
Depreciation				99,747,941	99,747,941	99,747,941	99,747,941	99,747,941						
Net Profit				-140,036,770	-59,014,149	-59,014,149	-59,014,149	-59,014,149	18,789,246	18,789,246	18,789,246	18,789,246	18,789,246	18,789,246
Cash Flow	-39,899,177	-299,243,824	-234,407,662	-40,288,829	40,733,793	40,733,793	40,733,793	40,733,793	18,789,246	18,789,246	18,789,246	18,789,246	18,789,246	18,789,246
CCF	-39,899,177	-339,143,001	-573,550,663	-80,188,005	-298,409,208	-257,675,415	-216,941,622	-176,207,830	-157,418,584	-138,629,338	-119,840,093	-101,050,847	-82,261,601	-63,472,356
DCF	-36,271,979	-247,308,946	-176,113,946	-27,517,812	25,292,481	22,993,164	20,902,876	19,002,615	7,968,474	7,244,068	6,585,516	5,986,833	5,442,575	4,947,796
DCCF	-36,271,979	-283,580,924	-459,694,871	-487,212,683	-258,288,444	-235,295,280	-214,392,403	-195,389,788	-187,421,314	-180,177,246	-173,591,730	-167,604,898	-162,162,323	-157,214,527

Table S5(a): Stream table showing most important process streams relating to Fig.2 in the main report. Flows are in kg/h.

	1	2	3	4	5	6	7	8	9	10	11	12	13
PET	6437.83	649.56											
LCC	12.86	12.86											
NaOH	1576.34												
H2SO4				23506.30				577.34	48.30				
Water	25082.45		25082.45		43889.76		4.38	1109.00	26.38				
EG			2654.25		2654.25	11.61	2609.40						
Na2TP			4710.37		0.01	0.01							
PTA					5550.30								
Na2SO4					3859.02	3859.02							
Cellulose								16654.05					
THF								242.32	242.32				
CaOH								399.70					
HMF										5462.53			
CaSO4									928.74				
Humins										10064.04			
Oxygen											1664.53		
AC												6061.48	
FDCA													6328.71
GVL													1.13

LCC= leaf compost cutinase, Na_2TP=sodium terephthalate, THF=tetrahydrofuran, AC=activated carbon, GVL=gamma-valerolactone

Table S5(b): Stream table showing most important process streams relating to Fig.2 in the main report. Flows are in kg/h.

	14	15	16	17	18	19	20	21	22	23	24	25	26
Water		9.93				978.03	3007	3311.64	33.27				
PTA											902.87	40.63	40.63
DMFD		47.33	6148.94										
Methanol	527	6.31								383.00	5.56		
PEF				7420.96	5550.30								
C. butirycum cells						5.51	2772.40	2772.40					
Fermentation media						2037.90							
Glycerol						6601.70							
FFA						81.50	81.50	81.50					
KCl						489.02	489.02		9.75				
PDO							3630.94	21.17	3609.77				
DMT											36.4	5347.97	
PTT													6667.27

FFA=free fatty acids

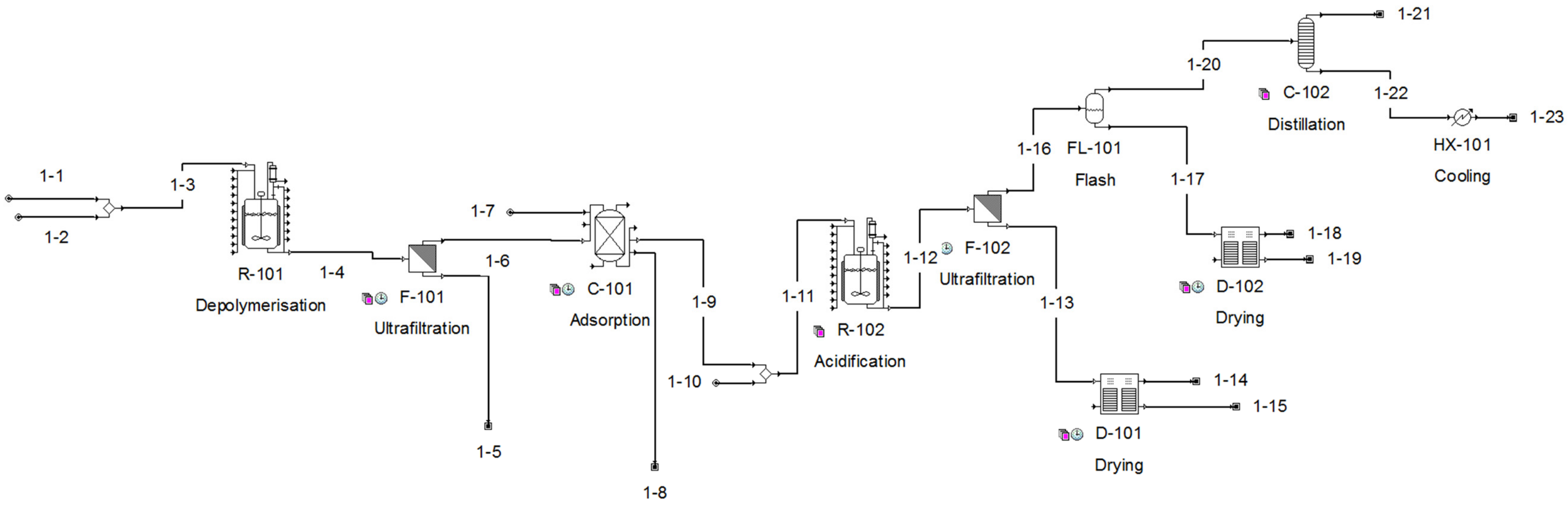

Figure S1(a): *Process flow diagram of Area 1 (PET depolymerisation). Stream 1-1: PET, stream 1-15: PTA, stream 1-19: Na_2SO_4, stream 1-23: EG.*

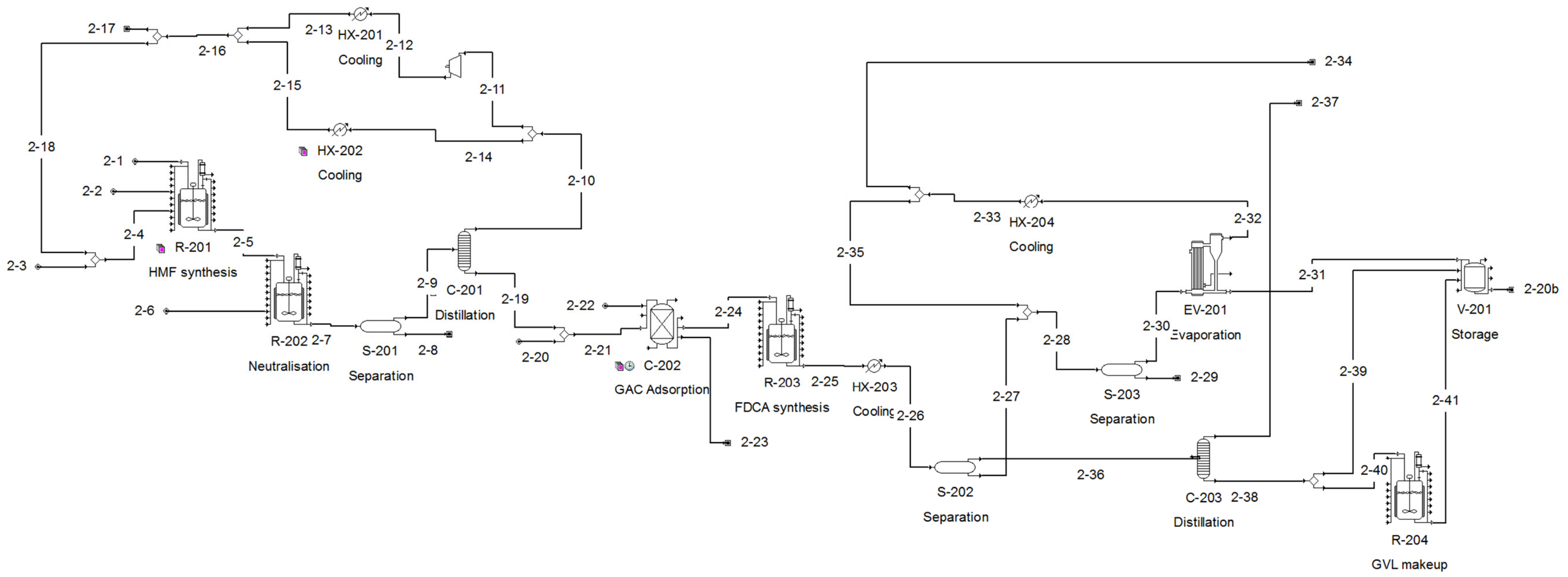

Figure S1(b): *Process flow diagram of Area 2 (FDCA production). Stream 2-1: cellulose, stream 2-29: FDCA, stream 2-20b: GVL recycle to stream 20 (not implemented due to limit in number of unit operations, but modelled in stream 2-20)*

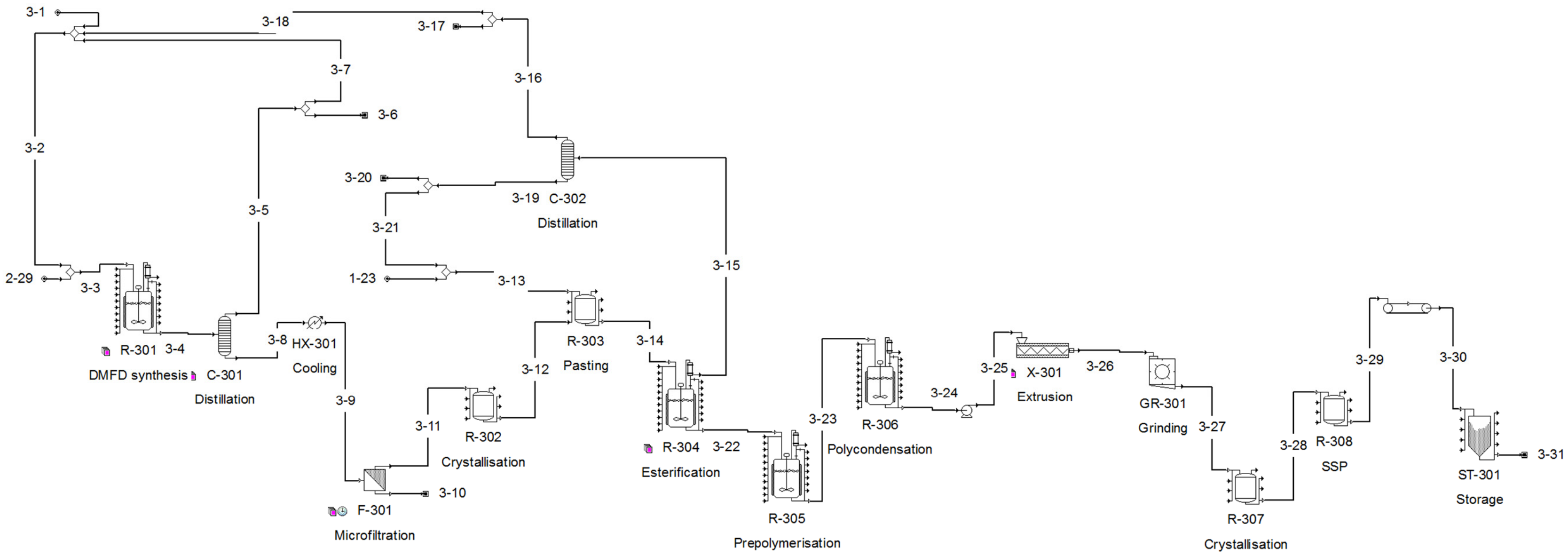

Figure S1(c): *Process flow diagram of Area 3 (PEF synthesis). Stream 2-29: FDCA, stream 3-1: methanol make-up, stream 1-23: EG, stream 3-31: PEF.*

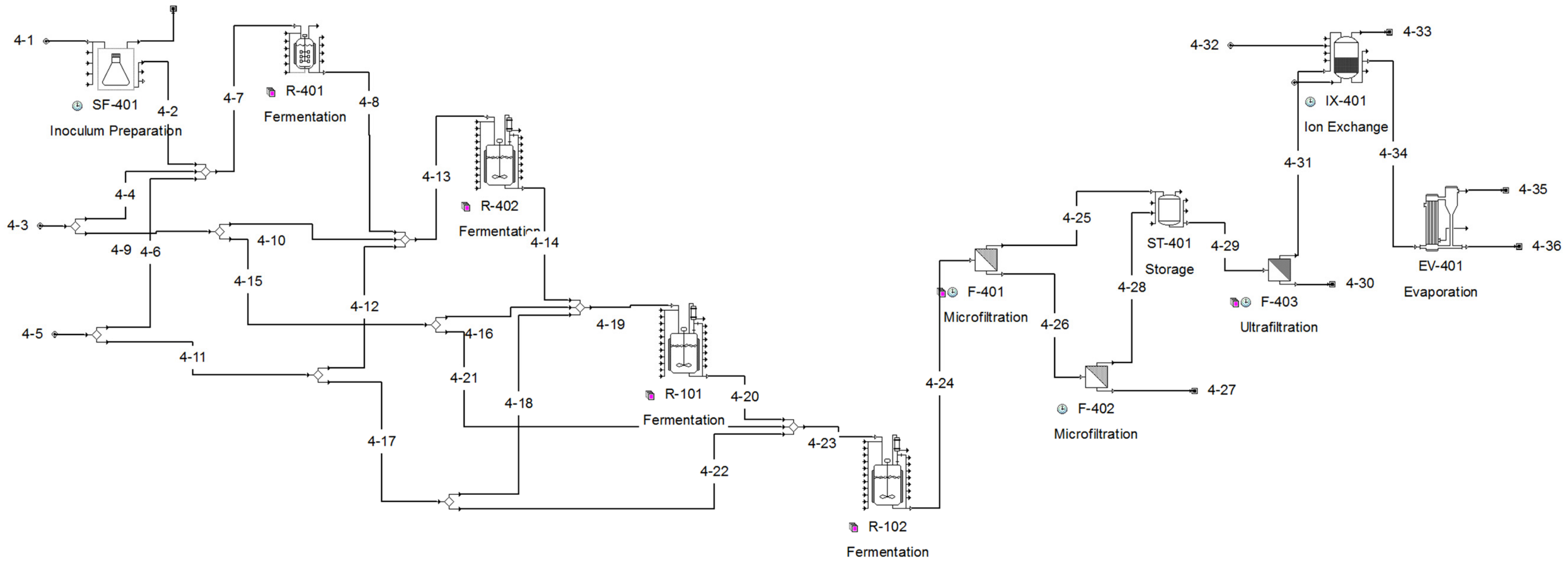

Figure S1(d): *Process flow diagram of Area 4 (PDO production). Stream 4-1: starter culture, stream 4-3: fermentation media, stream 4-5: crude glycerol, stream 4-36: PDO.*

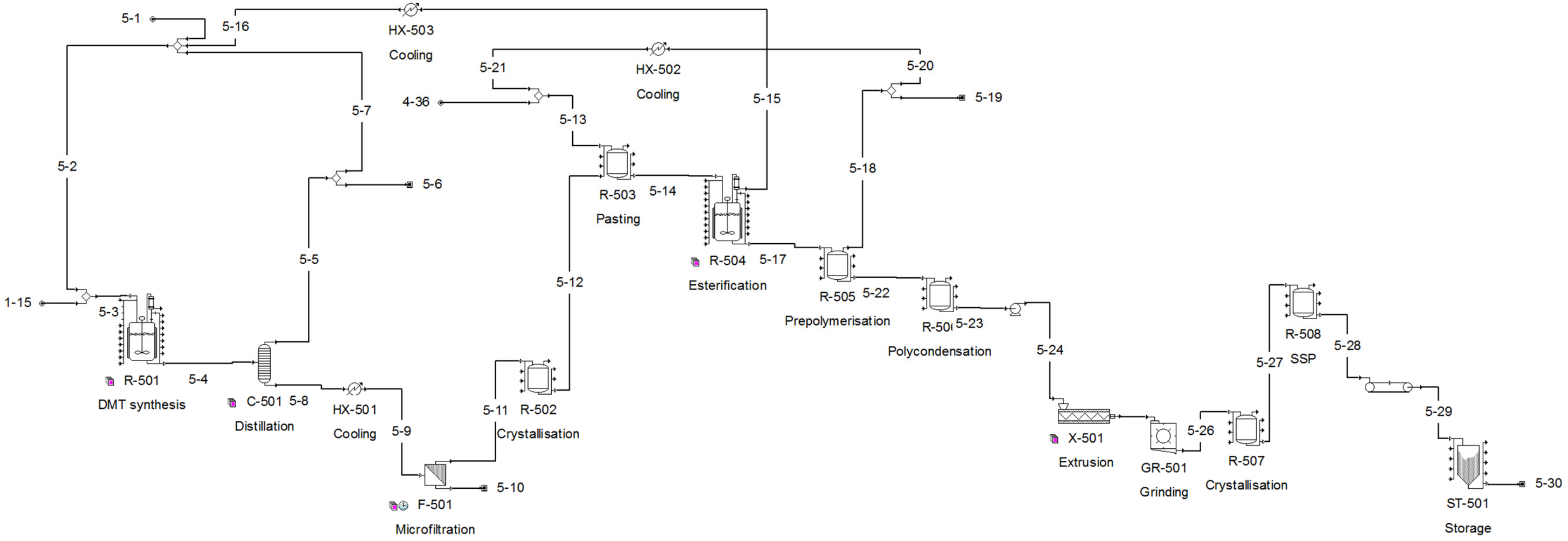

Figure S1(e): *Process flow diagram of Area 5 (PTT synthesis). Stream 1-15: PTA, stream 5-1: methanol make-up, stream 4-36: PDO, stream 5-30: PTT.*

References:

1. Tournier, V.; Topham, C. M.; Gilles, A.; David, B.; Folgoas, C.; Moya-Leclair, E.; Kamionka, E.; Desrousseaux, M. -L.; Texier, H.; Gavalda, S.; Cot, M.; Guémard, E.; Dalibey, M.; Nomme, J.; Cioci, G.; Barbe, S.; Chateau, M.; André, I.; Duquesne, S.; Marty, A. An engineered PET depolymerase to break down and recycle plastic bottles. *Nature* **2020,** *580*, pp. 216-219. doi:10.1038/s41586-020-2149-4.
2. Intelligen, Inc. SuperPro Designer Database, **2021.**
3. Kim, H.; Choi, J.; Park, J.; Won, W. Production of a sustainable and renewable biomass-derived monomer: conceptual process design and techno-economic analysis. *Green Chem.* **2020,** *22,* pp. 7070-7079. doi:10.1039/d0gc02258f.
4. Nomanbhay, S.; Hussein, R.; Ong, M. Y. Sustainability of biodiesel production in Malaysia by production of bio-oil from crude glycerol using microwave pyrolysis: a review**.** *Green Chemistry Letters and Reviews* **2018,** 11(2), pp. 135-157. doi: 10.1080/17518253.2018.1444795.
5. Public Health England, **2021**. *Bacteria Collection: Clostridium butyricum.* [Online]. Available at: https://www.phe-culturecollections.org.uk/products/bacteria/detail.jsp?refId=NCTC+7423&collection=nctc#medDoc. [Accessed 17 April 2021].
6. European Environment Agency, **2012**. *Agricultural, industrial and household water prices in late 1990s.* [Online]. Available at: https://www.eea.europa.eu/data-and- maps/figures/agricultural-industrial-and-household-water-prices-in-late-1990s. [Accessed 15 August 2021].
7. Sigma-Aldrich, **2021**. *Titanium(IV) butoxide*. [Online]. Available at: https://www.sigmaaldrich.com/catalog/product/aldrich/244112?lang=en&region= DK. [Accessed 17 August 2021].
8. Singh, A.; Rorrer, N. A.; Nicholson, S. R.; Erickson, E.; DesVeaux, J. S.; Avelino, A. F. T.; Lamers, P.; Bhatt, A.; Zhang, Y.; Avery, G.; Tao, L.; Pickford, A. R.; Carpenter, A. C.; McGeehan, J. E.; Beckham, G. T. Techno-economic, life-cycle, and socioeconomic impact analysis of enzymatic recycling of poly(ethylene terephthalate). *Joule* **2021,** 5, pp.1-25. doi:10.1016/j.joule.2021.06.015.
9. SalaryExplorer, **2021**. *Chemical Plant Operator Average Salary in Denmark 2021.* [Online]. Available at: http://www.salaryexplorer.com/salary- survey.php?loc=58&loctype=1&job=12174&jobtype=3. [Accessed 15 August 2021].
10. Davis, R.; Tao, L.; Tan, E. C. D.; Biddy, M. J.; Beckham, G. T.; Scarlata C.; Jacobsen, J.; Cafferty, K.; Ross, J.; Lukas, J.; Knorr, D.; Schoen, P. Process Design and Economics for the Conversion of Lignocellulosic Biomass to Hydrocarbons: Dilute-Acid and Enzymatic Deconstruction of Biomass to Sugars and Biological Conversion of Sugars to Hydrocarbons. National Renewable Energy Laboratory (NREL) Report, **2013,** NREL/TP-5100-60223.
11. Copenhagen Capacity, **2021**. *Taxation in Denmark.* [Online]. Available at: https://www.copcap.com/set-up-a-business/taxation. [Accessed 20 August 2021].
12. Seider, W. D.; Seader, J. D.; Lewin, D. R.; Widago, S. *Product and Process Design Principles: Synthesis, Analysis, and Evaluation*, 3rd ed.; John Wiley & Sons, Inc., United States of America, **2009**; pp. 534-627.
